# A Functional 2D Carbon Allotrope Combining Nanoporous Graphene and Biphenylene Segments

**DOI:** 10.1002/adma.202511706

**Published:** 2025-12-02

**Authors:** Paula Angulo‐Portugal, Martin Irizar, Longfeng Huang, Mamun Sarker, Mustafa A. Ashoush, Zakaria M. Abd El‐Fattah, Johannes Barth, Frederik Schiller, Afaf El‐Sayed, Fei Gao, Dimas G. de Oteyza, Alexander Sinitskii, Aran Garcia‐Lekue, Martina Corso, Ignacio Piquero‐Zulaica

**Affiliations:** ^1^ Centro de Física de Materiales (CFM‐MPC) CSIC‐UPV/EHU Manuel Lardizabal 5 San Sebastian 20018 Spain; ^2^ Department of Polymers and Advanced Materials: Physics, Chemistry and Technology Faculty of Chemistry University of the Basque Country UPV/EHU San Sebastian 20018 Spain; ^3^ Donostia International Physics Center (DIPC) Manuel de Lardizabal 4 San Sebastian E‐20018 Spain; ^4^ Physics Department E20 TUM School of Natural Sciences Technical University of Munich James‐Franck‐Straße 1 D‐85748 Garching Germany; ^5^ Department of Chemistry and Nebraska Center for Materials and Nanoscience University of Nebraska − Lincoln Lincoln Nebraska 68588 USA; ^6^ Physics Department Faculty of Science Al‐Azhar University Nasr City Cairo E‐11884 Egypt; ^7^ Physics Department Faculty of Science Galala University New Galala City Suez 43511 Egypt; ^8^ Department of Electrical and Computer Engineering University of California Los Angeles Los Angeles CA 90095 USA; ^9^ Nanomaterials and Nanotechnology Research Center (CINN) CSIC‐UNIOVI‐PA El Entrego 33940 Spain; ^10^ IKERBASQUE Basque Foundation for Science Plaza Euskadi 5 Bilbao 48009 Spain

**Keywords:** biphenylene, carbon allotropes, electronic structure, nanoporous graphene, on‐surface synthesis, photoemission spectroscopy, scanning probe microscopy

## Abstract

The implementation of graphene in semiconductor technology requires bandgap tuning, which can be achieved by chemical doping, geometric confinement in nanoribbons or with the insertion of nanopores and non‐hexagonal rings. The bottom‐up on‐surface synthesis approach in ultra‐high vacuum allows for the synthesis of atomically well‐defined graphene or biphenylene nanoribbons and nanoporous graphene (NPG) structures suitable for device applications. Here, a novel 2D carbon allotrope is synthesized in the form of a functional NPG with periodically spaced biphenylene segments. First, 12‐armchair porous graphene nanoribbons (12‐pGNRs) on Au(111) and Au(788) using 7,10‐dibromo‐1,4‐diphenyl‐triphenylene (DBDT) molecular precursor are grown. Low‐temperature scanning tunneling microscopy/spectroscopy (LT‐STM/STS) and non‐contact atomic force microscopy (nc‐AFM) measurements reveal the presence of high‐quality semiconducting 12‐pGNRs. Thermal annealing of densely packed 12‐pGNRs at 550 °C triggers their lateral fusion into diverse NPG structures featuring either graphene‐type or biphenylene‐type junctions. The structural and electronic properties are again characterized by LT‐STM and nc‐AFM in combination with density functional theory (DFT) calculations. DFT shows that while graphene‐type NPGs are direct bandgap semiconductors, biphenylene‐type NPGs manifest a smaller and indirect bandgap. The NPGs are stable upon oxygen and air exposure, and the nanopores feature a noticeable affinity to carbon monoxide, making them appealing systems for chemical sensors.

## Graphical abstract

The synthesis of a novel nanoporous graphene (NPG) is reported with biphenylene segments via thermal fusion of 12‐armchair porous graphene nanoribbons grown on gold surfaces. Characterization using STM, AFM, and DFT reveals low‐defect semiconducting behaviour and tunable band gaps. These NPGs are stable in ambient conditions and exhibit selective carbon monoxide affinity, making them promising for sensing applications.

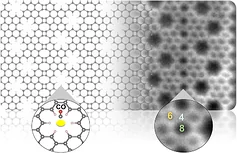

## 1 Introduction

Combining graphene^[^ ^1^ ^]^ with other low‐dimensional materials and molecules into hybrid systems has enabled the formation of layered materials with performance superior to that of the pristine components.^[^ ^2^ ^]^ Moreover, the incorporation of periodic non‐hexagonal rings into graphene can substantially modify its electronic and transport properties, leading to the creation of novel 2D carbon‐based materials.^[^ ^3^, ^4^, ^5^, ^6^ ^]^ In this respect, several 2D carbon allotropes with atomic topologies comprising non‐hexagonal carbon rings such as biphenylene, net‐graphene, and T‐graphene have been predicted theoretically.^[^ ^7^, ^8^, ^9^ ^]^ These extended systems exhibit metallic character, but both biphenylene and net‐graphene show anisotropic charge transport in contrast to the more isotropic T‐graphene.^[^ ^10^ ^]^ These novel 2D carbon allotropes are proposed to be suitable for electronics, optoelectronics, sensing,^[^ ^11^, ^12^ ^]^ and hydrogen^[^ ^13^ ^]^ or energy storage.^[^ ^14^, ^15^ ^]^ Using bottom‐up on‐surface synthesis strategies, biphenylene^[^ ^16^ ^]^ and net‐graphene^[^ ^17^ ^]^ nanoribbons, both featuring four, six, and eight‐membered carbon rings, were recently fabricated on noble metal surfaces. In the case of the former, even a semiconducting to metallic transition was demonstrated by increasing the nanoribbon's width.^[^ ^16^ ^]^

In addition to the introduction of non‐hexagonal carbon rings, the perforation of graphene with nanoscale pores leads to the formation of nanoporous graphene (NPG) structures, whereby the bandgap opens^[^ ^18^ ^]^ and a profound tuning of the electronic structure takes place depending on the pore's topology, size, and periodicity.^[^ ^19^, ^20^, ^21^, ^22^, ^23^ ^]^ Such on‐surface synthesized NPGs have recently been employed as prototype systems in field‐effect transistors (FETs)^[^ ^19^, ^24^ ^]^ and biosensors.^[^ ^25^ ^]^ However, merging these aforementioned 2D carbon‐based materials into a unique 2D hybrid material (i.e., combining NPGs and biphenylene together) remains a challenge due to the difficulty of embedding periodically non‐hexagonal rings and nanopores into a graphene lattice in a controllable way.

Here we report the synthesis of a novel 2D carbon allotrope comprising graphene, regularly spaced periodic nanopores and biphenylene stripes, thus potentially bringing together the emerging properties of NPGs and biphenylene. We use a bottom‐up synthesis approach to form this new material in a hierarchical fashion by employing 7,10‐dibromo‐1,4‐diphenyl‐triphenylene (DBDT) as a starting precursor molecule.^[^ ^26^, ^27^ ^]^ In contrast to previous works, where pores in graphene were achieved by lateral fusion of edge‐corrugated graphene nanoribbons,^[^ ^19^, ^20^, ^21^ ^]^ here, 12‐carbon‐atom‐wide graphene nanoribbons (GNRs) with embedded periodic nanopores and armchair edges spontaneously form through Ullmann‐like coupling of DBDT molecules and subsequent cyclodehydrogenation on a Au(111) surface. Additional annealing results in six distinct nanoribbon fusion patterns, exhibiting either graphene‐ or biphenylene‐type fusion. This process, therefore, yields the formation of diverse NPGs, frequently containing biphenylene lines. We demonstrate, with state‐of‐the‐art theoretical and experimental techniques, that the inclusion of biphenylene segments in the NPG reduces its bandgap and transforms it from a direct to an indirect semiconductor. Finally, we explore the response of the hybrid components to controlled carbon monoxide (CO) and oxygen (O_2_) gas dosing in ultra‐high vacuum (UHV) conditions. We observe that the nanopores show a significant affinity to CO but not to O_2_, foreseeing a possible use of this new semiconducting 2D carbon allotrope for selective gas sensing, supported by its robust stability in ambient conditions.

## 2 Results

**Figure 1:**
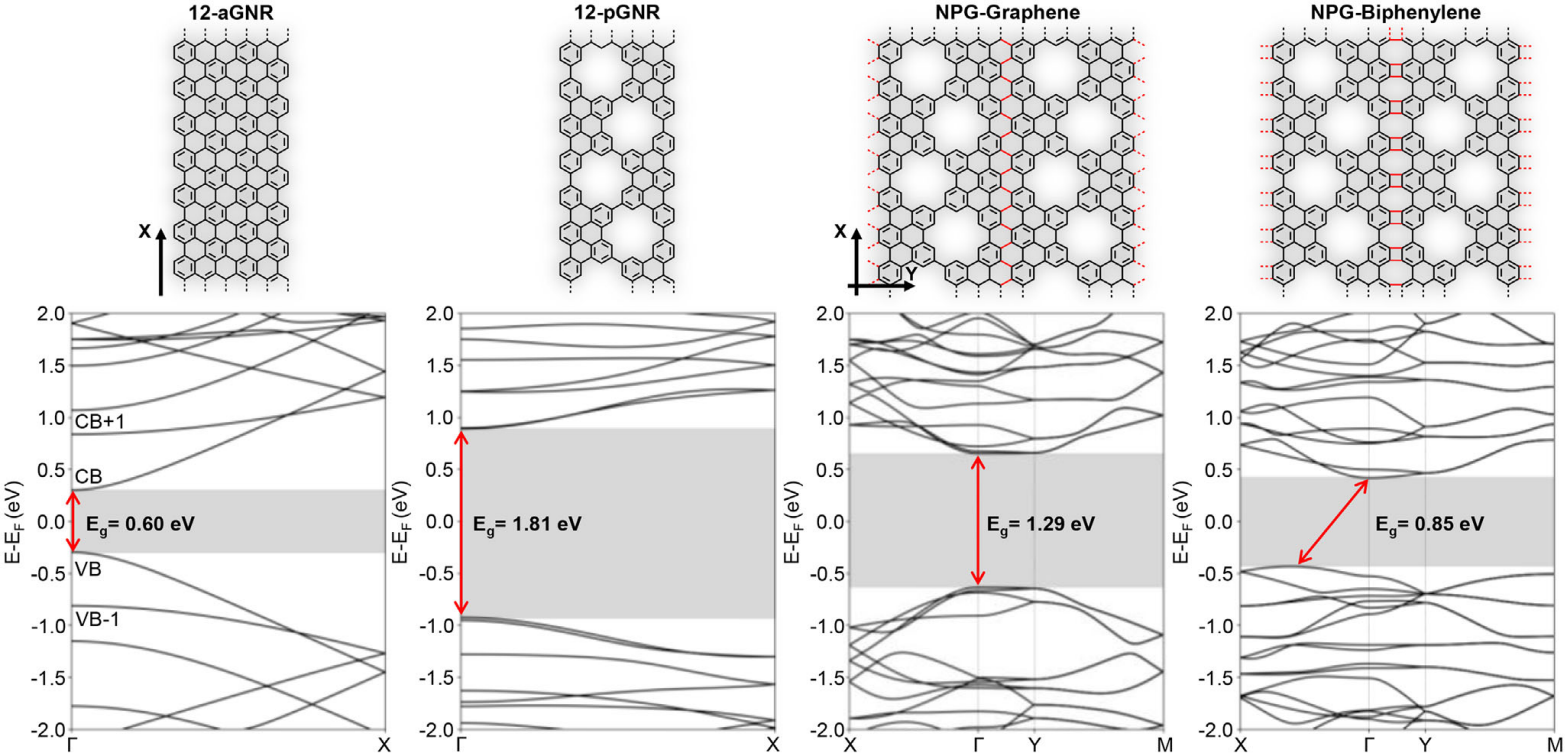
Structural models and DFT calculated band structures of 12‐aGNR, 12‐pGNR, NPG with a graphene‐type fusion pattern, and NPG with a biphenylene‐type fusion pattern. Bandgap values are indicated next to the red arrows denoting the direct or indirect transition. New bonds formed upon lateral fusion (resulting in 6‐ or 4‐ and 8‐membered rings) are highlighted in red. The abbreviations used (e.g., VB, CB, etc.) to identify the main frontier valence and conduction bands are also included in the 12‐aGNR band structure.

**Figure** **1** shows a comparative illustration of the atomic structures and the corresponding electronic band structures of the different carbon nanostructures studied in this work. Our density functional theory (DFT) calculations show that, upon perforation of a 12‐armchair graphene nanoribbon (12‐aGNR), the obtained porous armchair graphene nanoribbon (12‐pGNR) increases its bandgap about three times as compared to the pristine 12‐aGNR^[^ ^28^ ^]^ (i.e., from 0.6 to 1.81 eV). The 12‐pGNR is a 12‐carbon‐atom‐wide armchair GNR comprising neighboring [18]‐annulene nanopores. This is one of the smallest planar nanopore sizes one could consider in a graphene lattice with an effective diameter of ≈3.7 Å (extracted from the longest H to H distance within the nanopore). As a novelty, in this work, we propose that the lateral fusion of 12‐pGNRs into NPGs can occur in two ways, leading to the formation of either graphene‐like or biphenylene‐like lateral fusion patterns. The band structures of graphene‐type NPG (NPG‐G) and biphenylene‐type NPG (NPG‐BP) show not only a clear bandgap reduction with respect to 12‐pGNRs, but also a significant influence of the two different fusion patterns (i.e., graphene or biphenylene). While the extended NPG‐G exhibits a direct bandgap, the biphenylene fusion in NPG‐BP induces a stronger bandgap reduction as well as a transition to an indirect bandgap (see Figure 1).

**Figure 2:**
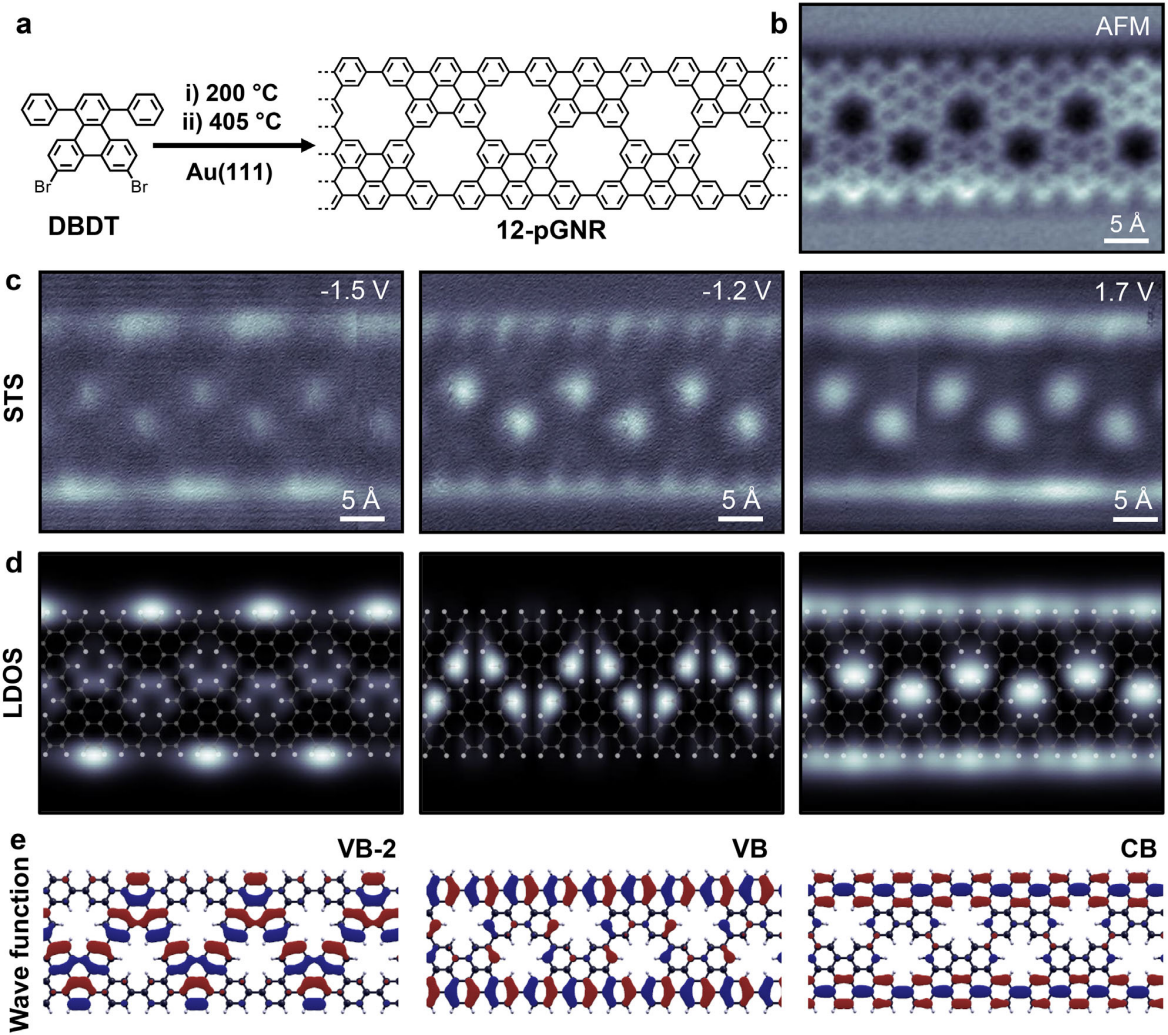
Synthesis and electronic structure of 12‐pGNRs. a) Schematic illustration of the synthesis of 12‐pGNRs starting from the DBDT precursor. b) nc‐AFM image of a 12‐pGNR on Au(111). c) Constant‐current (cc) d*I*/d*V* maps acquired with a CO‐functionalized tip at −1.5, −1.2, and 1.7 V. d) DFT‐based LDOS map simulations at energies close to the onsets of VB‐2, VB, and CB obtained at a height of *z*  =  6 Å away from the 12‐pGNR plane. e) Wave functions at the Γ point for VB‐2, VB, and CB.

**Figure** **2**a presents the synthesis diagram of 12‐pGNRs on Au(111) with the starting molecular precursor 7,10‐dibromo‐1,4‐diphenyl‐triphenylene (DBDT) and the final 12‐pGNR product. DBDT molecules, which were first synthesized in solution following a previously reported method,^[^ ^27^ ^]^ are then deposited on a clean Au(111) substrate held at room temperature (RT) in ultra‐high vacuum (UHV) conditions (see Experimental Section). An initial annealing step at 200 °C triggers the Ullmann‐like coupling reaction, producing DBDT‐polymers. A subsequent annealing step at 405 °C promotes C─C bond formation via cyclodehydrogenation, leading to the formation of 12‐pGNRs, as evidenced by nc‐AFM (Figure 2b; Figure S1, Supporting Information). The resulting final structure has an interpore periodicity of ≈12.7 Å, in good agreement with the previously published results on the same 12‐pGNRs.^[^ ^26^, ^27^ ^]^

The electronic structure of 12‐pGNRs was then characterized by LT‐STS. Since the molecular orbitals are spatially inhomogeneous,^[^ ^20^ ^]^ we first performed STS at different positions of the 12‐pGNR (as shown in Figure S2, Supporting Information). In agreement with Fan et al.,^[^ ^26^ ^]^ we find the main resonances of the occupied states and unoccupied states at −1.5 and 1.7 V, respectively, which are more clearly observed at the edges of the ribbon (see Figure S2, Supporting Information). Figure 2c shows the measured constant current (cc) d*I*/d*V* maps at −1.5 and 1.7 V, which, as expected, exhibit strong conductance features confined to the nanopores and nanoribbon edges.^[^ ^20^ ^]^ However, upon comparison with calculated LDOS maps and wave functions (Figure 2d,e), we observe that the map acquired at −1.5 V corresponds to the VB‐2, and not to the frontier valence bands (VBs).^[^ ^26^ ^]^ Instead, the d*I*/d*V* map taken at −1.2 V (i.e., closer to the Fermi level) shows a different symmetry which, indeed, corresponds to the quasi‐degenerate VB and VB‐1 bands (see Figures S2 and S3, Supporting Information for the identification of the frontier bands). For the unoccupied states, the frontier CBs (i.e., the degenerate CB and CB+1) are identified at 1.7 V. The striking difference between the experimental d*I*/d*V* maps (or the calculated LDOS at 6 Å above the carbon backbone) with respect to their corresponding wave functions is attributed to the well‐known decay of the wave functions into the vacuum^[^ ^20^, ^29^ ^]^ (see Figure S4, Supporting Information). As a consequence, in the experiment, conductance features appear confined along the GNR edges and inside the small nanopores. Note that the intensity inside the nanopores does not correspond to the confinement of the Au(111) surface state^[^ ^30^, ^31^ ^]^ since the LDOS maps were calculated in the free‐standing configuration. Additional cc d*I*/d*V* maps are included in Figures S5 and S6 (Supporting Information). Given that the frontier bands are now identified ≈−1.2 and 1.7 V, respectively, we obtain an experimental electronic bandgap on Au(111) of ≈2.9 V, almost three times larger than the reported 1.13 V bandgap for the non‐porous pristine 12‐aGNRs.^[^ ^32^ ^]^ This is in agreement with the DFT band structure trend shown in Figure 1 and with previously published DFT simulations on 12‐pGNRs.^[^ ^26^, ^28^ ^]^ Note that the screening effect of Au(111) reduces the bandgap of the isolated 12‐pGNRs.^[^ ^33^, ^34^ ^]^ Overall, the agreement between d*I*/d*V* maps and free‐standing LDOS simulations clearly suggests a weak 12‐pGNR‐substrate interaction.

**Figure 3:**
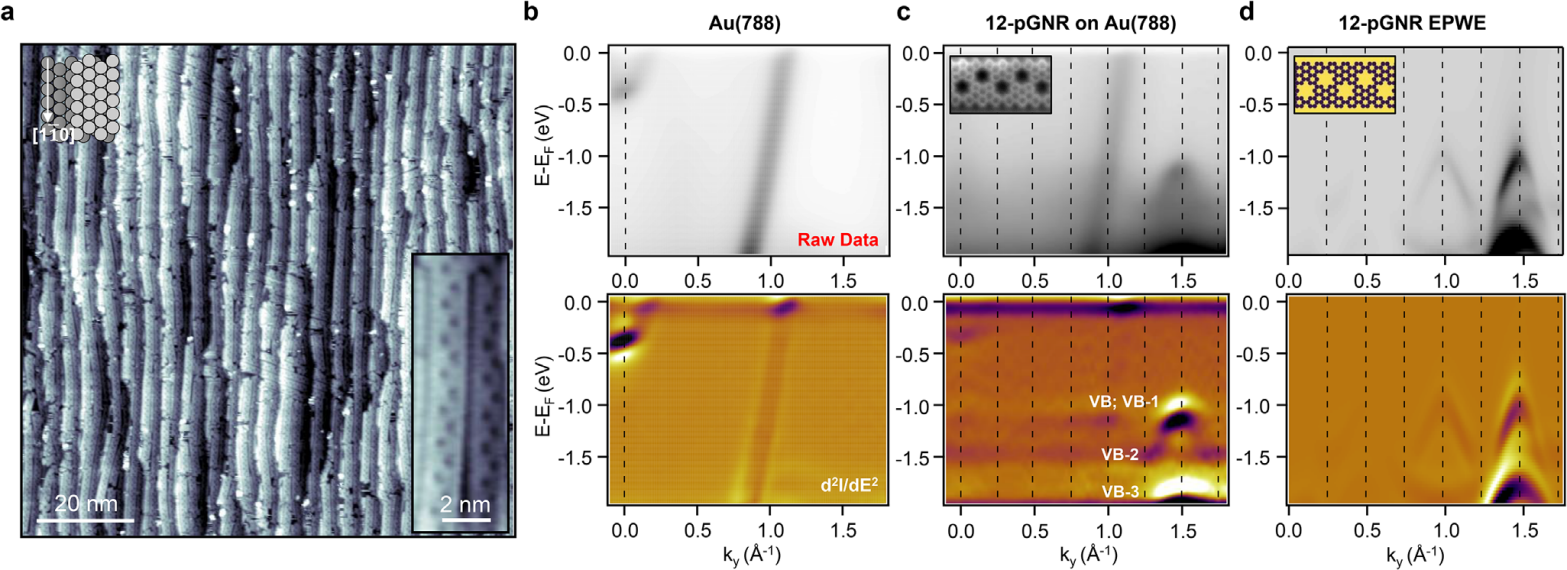
Structure and electronic properties of 12‐pGNRs on Au(788). a) RT‐STM of 12‐pGNRs grown on Au(788) with a zoom‐in (bottom‐right) showing the preferential alignment of 12‐pGNRs. STM parameters: *V*  =  −1.2 V; *I*  =  50 pA. Inset (top‐left) shows the atomic arrangement of the substrate steps. b, c) Photoemission intensity maps in raw data (top) and second derivative (bottom) for the pristine and 12‐pGNR covered Au(788). d) EPWE simulations of 12‐pGNRs without substrate.

To further explore the occupied electronic band structure of 12‐pGNRs, we performed angle‐resolved photoemission spectroscopy (ARPES) measurements. Since the growth of 12‐pGNRs on Au(111) preferentially occurs along the three high symmetry crystallographic directions, we used different stepped surfaces, namely, kinked step Au(13 11 12) and straight step Au(788) surfaces, in order to align the 12‐pGNRs along the steps (see Figure S7, Supporting Information). The best 12‐pGNR alignment was found on Au(788), where 12‐pGNRs preferentially align along the steps in the close‐packed [1‐10] direction (**Figure** **3**a). We stress that this uniaxial alignment of 12‐pGNRs is crucial for the following ARPES measurements^[^ ^35^, ^36^, ^37^, ^38^ ^]^ (see Experimental Section).

In Figure 3b,c, we show ARPES photoemission intensity maps acquired along the step direction of Au(788) for the pristine surface (Figure 3b) and for the surface fully covered with 12‐pGNRs (Figure 3c). The photoemission intensity is shown in raw data (top) and second derivative (bottom), the latter of which is used to enhance the spectral features and improve the visualization of the bands. For the pristine Au(788) case, the Au surface state parabola is identified at the $\bar{\Gamma }$ point, and the highly dispersive sp‐band crossing the Fermi energy appears at higher momentum. For the 12‐pGNR case, new weakly dispersive bands emerge ≈1.5 Å^−1^. The second derivative plot clearly highlights the weak dispersions and replicas of the main frontier bands. The vertical dashed lines indicate the Brillouin zone centers and boundaries that repeat every $\frac{2\pi }{a}$, where *a* is the periodicity of the 12‐pGNR (i.e., ≈12.7 Å). Electron Plane Wave Expansion (EPWE)^[^ ^39^ ^]^ band structure and photoemission intensity map simulations (Figure 3d) allow us to properly identify the different VBs in the second derivative of the experimental data (see also Figure S8, Supporting Information). We assign the first dispersive band at −1.1 eV to the quasi‐degenerate VB and VB‐1. VB‐2 has very weak spectral weight in normal emission, but can still be discerned at −1.5 eV.^[^ ^40^, ^41^ ^]^ The third weakly dispersive band onset at −2.0 eV corresponds to VB‐3. The assumption of a weak interaction between 12‐pGNRs and Au(111) or Au(788) is further supported by the resemblance of the ARPES bands with the free‐standing EPWE simulations (see Figure 3c,d). In summary, the complementary ARPES and STS techniques clearly identify the frontier VBs that lie at similar energies.

**Figure 4:**
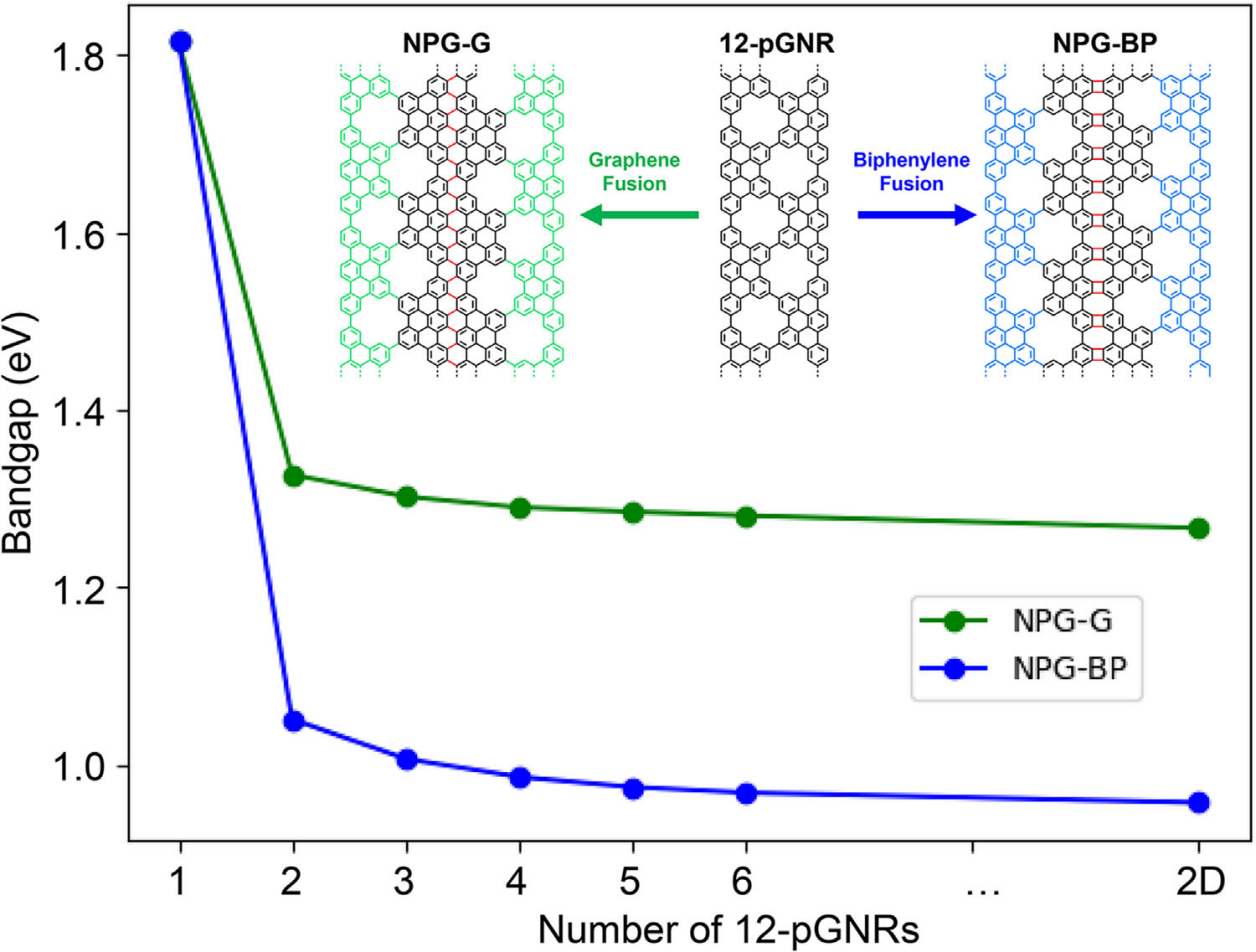
DFT calculated evolution of the bandgap for graphene‐type and biphenylene‐type NPGs, starting from the 12‐pGNR up to the 2D extended system. For both systems, a clear bandgap reduction takes place already for two fused 12‐pGNRs. This bandgap remains almost unchanged up to the extended limit. Such abrupt bandgap reduction is related to the formation of chevron nanoribbons between the nanopores (highlighted in black in the structural models). Once these are formed, the weak inter‐ribbon coupling does not substantially alter the bandgap.

The low defect density in the formation of 12‐pGNRs (see Figures S9a and S10, Supporting Information) enables the exploration of NPG formation by annealing a 12‐pGNR fully‐covered Au(111) substrate at 550 °C. We observe a successful lateral fusion of 12‐pGNRs into different NPG structures, whereby NPGs composed by 2 to 6 fused 12‐pGNRs are recurrently observed (Figure S9, Supporting Information). A description of the most abundant defects encountered is given in Figure S10 (Supporting Information). Previous STM studies on these NPGs could only identify two graphene‐type lateral fusion configurations,^[^ ^27^ ^]^ however our extensive nc‐AFM measurements reveal six possible lateral fusion configurations on the Au(111) surface (Figure S11, Supporting Information) that yield four distinct NPG structures (Figure S12, Supporting Information): two NPGs with graphene type fusion (NPG‐G) and another two with biphenylene type fusion (NPG‐BP). While the graphene fusion pattern gives rise to a chevron type GNR^[^ ^42^ ^]^ between the pore arrays (highlighted in black in **Figure** **4**), (c‐GNR), the biphenylene fusion leads to a chevron biphenylene nanoribbon (c‐BPR) between the pore arrays. These experimental nc‐AFM observations (Figure S11, Supporting Information) confirm our initial hypothesis presented in Figure 1 and validate the on‐surface synthesis of NPG‐G and NPG‐BP. For clarity, the structural models of the different NPGs are summarized in Figure S12 (Supporting Information). Remarkably, NPG‐BP represents a novel 2D carbon allotrope (i.e., comprising graphene, periodically spaced nanopores, and biphenylene segments) that has never been achieved before, and it is clearly distinct to other reported direct‐bandgap 2D carbon allotropes such as graphdiyne and graphyne (with *sp* and *sp^2^* hybridized carbon atoms).^[^ ^5^, ^6^ ^]^

First, DFT band structure calculations are performed on NPG‐G and NPG‐BP to analyze the evolution of the bandgap with increasing number of fused 12‐pGNRs. The results, summarized in Figure 4, reveal that regardless of the fusion pattern (i.e., graphene or biphenylene), the bandgap undergoes a drastic reduction upon fusion of two 12‐pGNRs, but shows only minor changes henceforth until the fully extended 2D framework is reached. The difference between the initial and the following fusion events is that the former results in the formation of the aforementioned chevron nanoribbon embedded between the pores, whereas all the next fusion steps only incorporate additional laterally coupled chevron nanoribbons. From this, we can infer that i) it is the c‐GNR and c‐BPR segments which mainly define the electronic properties of NPG‐G and NPG‐BP, respectively, and ii) that the chevron nanoribbons are only weakly coupled in the NPGs, and thus hardly change the NPG bandgap with increasing number of fused 12‐pGNRs.

**Figure 5:**
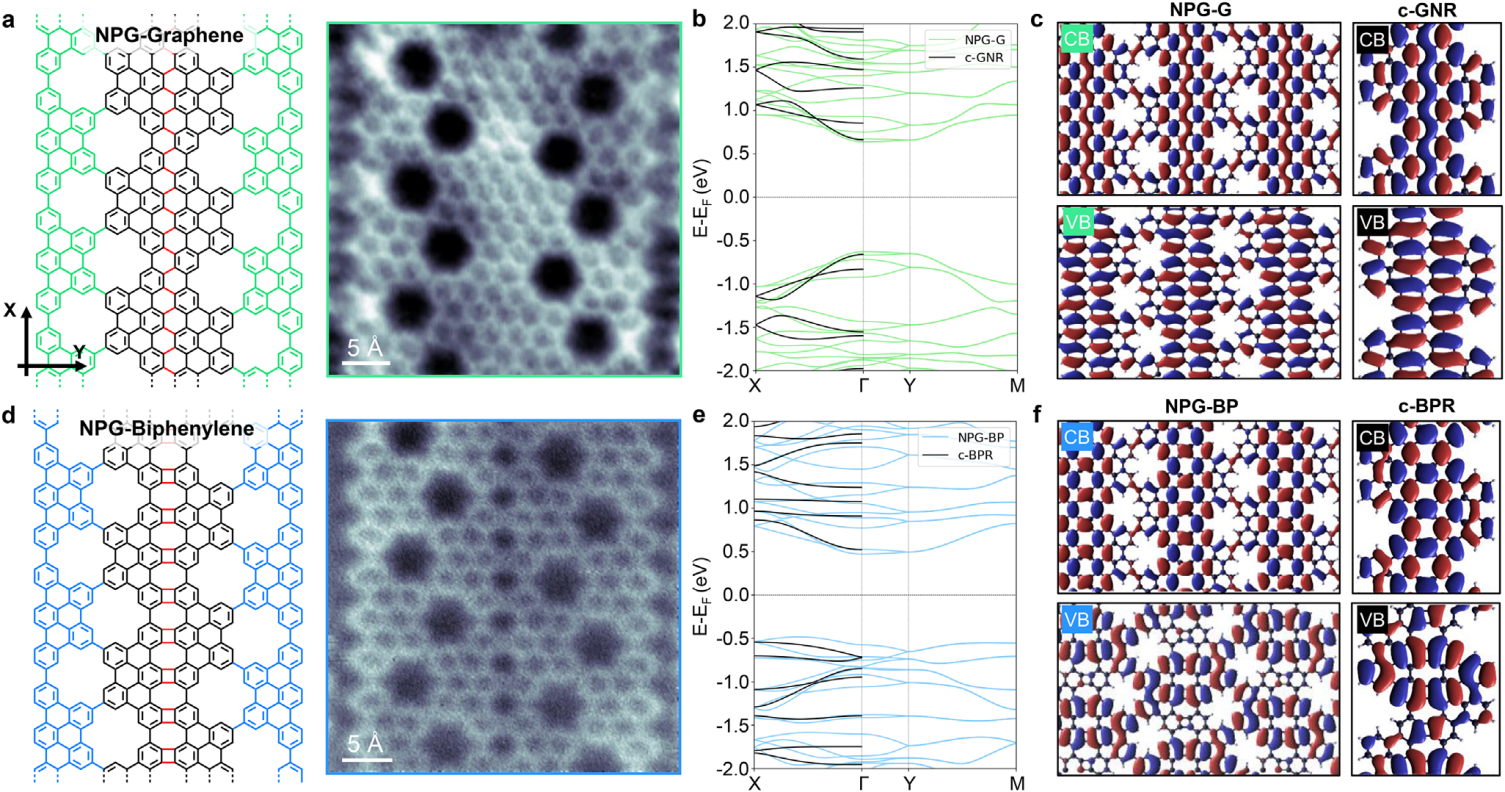
Structure and electronic properties of graphene and biphenylene‐type NPGs and comparison with their chevron nanoribbon building blocks. a) Schematic model and nc‐AFM image of NPG‐G. The c‐GNR segment is highlighted in black, and the graphene type fusion in red. b) DFT calculated electronic band structure of NPG‐G (green) and c‐GNR (black). c) NPG‐G and c‐GNR frontier band wave functions at the Γ point. d) Schematic model and nc‐AFM image of NPG‐BP. The c‐BPR segment is highlighted in black, and the biphenylene type fusion in red. e) DFT electronic band structure of NPG‐BP (blue) and c‐BPR (black). f) NPG‐BP and c‐BPR frontier band wave functions at the Γ point.

Further evidence of the direct interrelation between the electronic properties of c‐GNR and c‐BPR with NPG‐G and NPG‐BP, respectively, is given in **Figure** **5**. Figure 5a shows the structural model and nc‐AFM image of NPG‐G. Figure 5b presents the band structure of the NPG‐G (in green), compared to the band structure of the c‐GNR segment (in black). Indeed, we clearly see that the band dispersion of NPG‐G along the ΓX direction (i.e., along the armchair direction of the NPG) resembles that of the c‐GNR (in black) and not that one of the 12‐pGNR (see Figure 1), proving that the electronic structure of NPG‐G is governed by the c‐GNR segment defined upon the lateral fusion of 12‐pGNRs (Figure 5a). This is further supported when comparing the frontier band wave functions in Figure 5c, where both the NPG‐G VB and CB wave functions at the Γ point show the same pattern and symmetry as the c‐GNR ones. In addition, a weak orbital amplitude around the bonds where c‐GNRs couple together supports the low inter‐ribbon coupling. This is also evidenced by the noticeable loss of band dispersion in the ΓY direction (i.e., along the zig‐zag direction of the NPG‐G, see Figure 5a,b), reflecting the highly anisotropic electronic structure of NPG‐G.^[^ ^19^, ^20^ ^]^

Moving on to the biphenylene fusion case, Figure 5d shows the structural model and nc‐AFM of NPG‐BP. Here, a c‐BPR arises when two 12‐pGNRs fuse together as highlighted in black in the model. The band structure of NPG‐BP is presented in Figure 5e (in blue). Compared to NPG‐G, a clearly smaller bandgap becomes evident (1.27 vs 0.95 eV), as well as a change from a direct bandgap to an indirect one (see ΓX direction). Both of these changes are readily apparent when comparing c‐GNR and c‐BPR (see black bands), confirming that the NPG‐BP band structure does not directly relate to that of the 12‐pGNRs (see Figure 1) but rather to that of c‐BPR. This is further supported by looking at the frontier band wave functions shown in Figure 5f. The pattern and symmetry of the VB and CB wave functions at the Γ point of NPG‐BP are again similar to the c‐BPR ones (see Figure S13, Supporting Information). For completeness, the structural models, DFT band structure, and wave function calculations for the four distinct NPG structures are summarized in Figures S12 and S14 (Supporting Information). Note that in the experiment, we also observe hybrid NPG structures containing both graphene and biphenylene segments (see Figure S9d, Supporting Information). As a way of example, we calculated the DFT band structures of two hybrid NPG‐G‐BP configurations (i.e., alternating graphene and biphenylene fusion) and observed that the frontier bands are dominated by the c‐BPR segments and the indirect‐bandgap character is still preserved (see Figure S15, Supporting Information).

Beyond this detailed electronic structure characterization, we also studied the mechanical properties of NPG‐G and NPG‐BP by calculating stress‐strain curves with DFT (see Figure S16, Supporting Information). We observe that, in comparison to pristine graphene,^[^ ^43^ ^]^ the inclusion of nanopores and biphenylene segments into the NPG leads to a strong reduction of the Young's modulus. The calculated values for NPG‐G and NPG‐BP are comparable to those previously reported for other NPG structures.^[^ ^44^, ^45^ ^]^ More interestingly, our results show that the biphenylene units in NPG‐BP induce a drastic quenching of the mechanical anisotropy observed in NPG‐G (Figure S16, Supporting Information).

**Figure 6:**
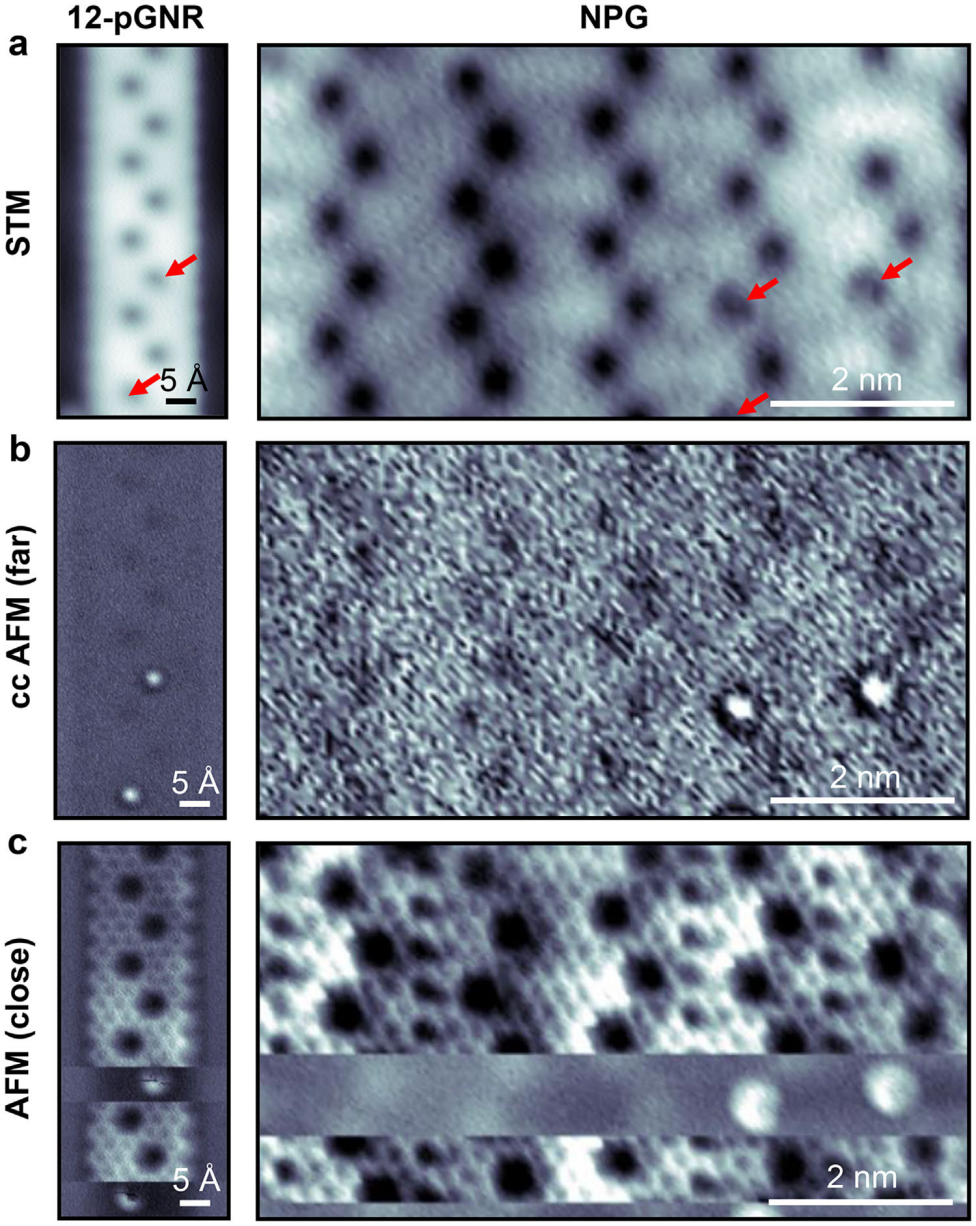
Preferential CO adsorption in 12‐pGNR and NPG nanopores. Simultaneously acquired a) LT‐STM and b) cc nc‐AFM images of 12‐pGNR (left) and NPG (right) at 6 K. STM parameters: *V*  =  50 mV; *I*  =  10 pA. c) Bond‐resolved constant‐height nc‐AFM images. Here, the tip was retracted by 1 Å above the CO‐functionalized nanopores.

The incorporation of nanopores and biphenylene segments into graphene is also expected to enhance the reactivity of these novel 2D carbon‐based materials, making them suitable for sensing and sieving applications.^[^ ^11^, ^46^, ^47^, ^48^, ^49^ ^]^ With this in mind, we studied the functionality of 12‐pGNRs and NPGs at low temperature as shown in **Figure** **6**. After dosing 1.8 L of CO on samples held at 6 K (see Experimental Section), LT‐STM measurements reveal only a very weak contrast change in certain pores (highlighted with red arrows in Figure 6a). AFM measurements acquired simultaneously with STM (i.e., in cc mode) show a clear frequency shift variation, producing a circular pattern in these nanopores (Figure 6b), suggesting the presence of CO molecules trapped within the nanopores. To unambiguously verify this hypothesis, we performed bond‐resolved nc‐AFM (Figure 6c). Here, we obtain bond‐resolved images of both 12‐pGNRs and NPGs in the regions without any trapped CO molecule. As we approach the functionalized pores, the tip is lifted by 1 Å to avoid strong interactions with the CO molecules (i.e., the excessive repulsive interaction between the CO at the tip and the one on the pore at close distance can induce the movement of the trapped CO away from the pore). Indeed, a bright circle is still observed on the functionalized pores, confirming the presence of CO molecules. Due to the small diameter of the nanopores and the presence of the H atoms decorating them, the CO molecules cannot enter or pass through the nanopores and remain physisorbed on the NPG. Scanning at high voltages (≈3 V) usually triggered the hopping of the COs from pore to pore, suggesting a weak but noticeable CO‐pore interaction. Note that no CO molecule was observed on the biphenylene segments (for this particular CO dose and sample temperature), suggesting a lower CO‐affinity than the nanopores.

The realization of GNR and NPG‐based electronic devices, such as FETs requires either the direct growth of GNRs and NPGs on technologically relevant substrates or the transfer of such low‐dimensional graphene‐based materials from metallic substrates to insulating substrates (such as SiO_2_, HfO_2,_ or Al_2_O_3_). Currently, the transfer from Au films to oxides by chemical etching is the most widely used strategy to bring GNRs and NPGs on suitable substrates for FETs nanofabrication.^[^ ^19^, ^24^, ^50^, ^51^ ^]^ Based on such works, we consider that the 12‐pGNRs and NPG structures reported here are similarly suitable for device fabrication. To validate this hypothesis, we tested the stability of the 12‐pGNRs and NPGs upon oxygen and air exposure. Armchair GNRs have been demonstrated to be stable in air.^[^ ^52^ ^]^ However, the [18]‐annulene nanopores contain zig‐zag edges, the latter of which are known to be reactive when exposed to air.^[^ ^53^ ^]^ In order to test their robustness in oxidizing environments, we first exposed the samples to 450 L of O_2_ in UHV (see Experimental Section). The nc‐AFM images and cc d*I*/d*V* maps (Figure S17, Supporting Information) show neither structural nor electronic changes compared to the unexposed 12‐pGNRs (cf. Figures S5 and S6, Supporting Information). In addition, we exposed 12‐pGNRs and NPGs to air (for 1h). Combined X‐ray photoelectron spectroscopy (XPS) and RT‐STM measurements demonstrate that the materials are stable in air, which represents the first step toward scalability (see Figure S18, Supporting Information). These findings highlight 12‐pGNRs and NPGs as promising platforms featuring nanopores that could serve as effective gas‐trapping active sites for sensing purposes.

## 3 Conclusion

In this work, we show the on‐surface synthesis of a novel 2D carbon allotrope, namely an NPG featuring periodically spaced nanopores and biphenylene segments. This structure was achieved through a hierarchical on‐surface synthesis process involving the initial formation of 12‐pGNRs followed by their lateral fusion into NPGs. First, we confirmed the ≈2.9 eV wide bandgap semiconducting character of 12‐pGNRs using STS and ARPES. We then explored the lateral fusion of 12‐pGNRs, which induces the formation of NPGs with graphene‐type or biphenylene‐type lateral fusion patterns. Both NPG types feature a notable bandgap renormalization and pronounced electronic anisotropy. Specifically, NPG‐G shows a direct bandgap, whereas NPG‐BP exhibits a smaller and indirect bandgap. The electronic properties of such NPGs are governed by the chevron nanoribbon segments formed during the lateral fusion of 12‐pGNRs. These direct or indirect bandgap materials may be suitable for their implementation in light‐emitting, solar cells, or other electronic devices. Finally, both 12‐pGNRs and NPGs exhibit stability upon exposure to oxygen and air, as well as a strong affinity to CO in the nanopores, highlighting their potential use in chemical sensing applications.

## 4 Experimental Section

### On‐Surface Synthesis

Au(111), Au(788), and curved Au(645) crystals were cleaned via Ar^+^ sputtering and annealing cycles ranging between 450 and 550 °C. DBDT molecules were sublimed from a Knudsen cell at 180 °C and deposited on the corresponding gold substrates held at room temperature in UHV conditions. Then, the samples were annealed at 200 °C for 10 min to form the DBDT polymers and at 405 °C for 20 min to form 12‐pGNRs. An additional annealing step at 550 °C for 1 min or at 530 °C for 30 min [with the Au(111) surface fully‐covered by 12‐pGNRs] was required to obtain the NPGs.

### Gas Exposure

CO was deposited on the sample by opening the LT‐STM shields and dosing 1.8 L (2 × 10^−8^ mbar of CO for 2 min) in the LT‐STM chamber while keeping the sample between 6 and 10 K. O_2_ exposure was carried out in the preparation chamber by dosing 450 L of O_2_ gas (1 × 10^−6^ mbar for 10 min) on samples kept at room temperature.

### RT‐STM Characterization

RT‐STM measurements were performed with a commercial Scienta‐Omicron VT‐STM set‐up at room temperature. WSxM^[^ ^54^ ^]^ software was used to process STM images.

### LT‐STM, nc‐AFM, and STS Characterization

LT‐STM, nc‐AFM, and STS measurements taken with a CO‐functionalized tip were performed with a CreaTec STM/AFM instrument in UHV conditions (*p* ≈10^−10^ mbar) at a temperature of *T* = 6 K. STM images were recorded in constant current (cc) mode. nc‐AFM imaging was performed in frequency modulation mode using a qPlus sensor (resonance frequency ≈30 kHz, Q value > 11000, *k* ≈1800 Nm^−1^, oscillation amplitude of 80 pm). AFM scans were acquired at constant height (i.e., with the *z*‐feedback switched off) at a sample bias of *V*  =  0 V with CO‐functionalized W tips. CO‐functionalized tips provided enhanced resolution in both STM topographic images and d*I*/d*V* maps. d*I*/d*V* measurements were recorded using a lock‐in amplifier with modulation frequency and voltage (*V*_mod (0‐to‐peak)_) of 403 Hz and 30 mV, respectively. d*I*/d*V* point spectra were recorded under open feedback loop conditions. STM images were subjected to standard corrections, such as plane‐subtraction and global brightness/contrast adjustments. The images were processed using WSxM^[^ ^54^ ^]^ and SpmImage Tycoon.^[^ ^55^ ^]^

### Angle Resolved Photoelectron Spectroscopy (ARPES)

ARPES measurements were performed with a laboratory‐based experimental setup using a display‐type hemispherical electron analyzer (SPECS Phoibos 150, energy/angle resolution of 40 meV/0.1°) combined with a monochromatized Helium I_α_ (hν = 21.2 eV) source. Measurements were acquired with the sample at 130 K for 12‐pGNRs on Au(788) and at room temperature for the pristine Au(788) sample, by moving the polar angle, which was set to be parallel to the steps and along the $\bar{\Gamma K}$ direction of Au(111) terraces.

### X‐Ray Photoelectron Spectroscopy (XPS)

X‐ray light (monochromatized Al K_α_, hν = 1486.6 eV) was produced from a Specs microfocus setup µFOCUS600. The experimental resolution was determined to be 500 meV. Measurements were acquired with the sample at room temperature at 65° incidence and 40° emission angle. All energy positions were referred to the Fermi level measured on the same sample under the same experimental conditions.

### Density Functional Theory

First‐principles calculations were completed using Density Functional Theory (DFT) as implemented in the SIESTA package.^[^ ^56^, ^57^, ^58^ ^]^ Exchange‐correlation was treated via the van der Waals (vdW) density functional by Dion et al.,^[^ ^59^ ^]^ reparametrized by Klimeš, Bowler, and Michaelides.^[^ ^60^ ^]^ The core electrons have been described by the norm‐conserving Troullier‐Martins pseudopotentials^[^ ^61^ ^]^ and generated with the ATOM package included in the SIESTA software, the cutoff radii being (all in bohr units) 1.01, 1.28, 1.60 and 1.60 for the s, p, d, and f channels of C; and 1.31, 1.13, 1.09 and 1.14 for H. The unit cell of the 12‐pGNR has a relaxed lattice parameter of a = 13.12 Å in the periodic direction to the ribbon, and b = 50 Å and c = 20 Å in the other in‐plane and out‐of‐plane directions to avoid interactions with neighboring cells. The unit cells of the 2D structures were obtained by merging 12‐pGNRs laterally, where the exact lattice parameters depend on the type of lateral fusion. The reciprocal spaces have been discretized employing a Monkhorst‐Pack k‐grid of 8 × 1 × 1 and 8 × 4 × 1 for 1D and 2D structures, respectively. The structures were allowed to relax up to a force tolerance of 10 meV/Å. Scanning Tunneling Microscopy (STM) simulations were performed using a SIESTA utility, called STM, which was based on the Tersoff‐Hamman approximation.^[^ ^62^ ^]^

The mechanical properties of the NPGs, i.e., the stress–strain curves, were evaluated through a series of DFT calculations using the same computational parameters as described previously. The uniaxial stress‐strain behavior was examined for NPGs elongated along the zigzag and armchair directions, respectively. The supercell stress calculated with SIESTA was rescaled by c/d0, where c represents the size of the supercell in the direction perpendicular to the NPG (20 Å in this work) and d0 is the interlayer distance of graphite.^[^ ^63^ ^]^

## Conflict of Interest

The authors declare no conflict of interest.

## Author Contributions

P.A.‐P. and M.I. contributed equally to this work. I.P.‐Z. and M.C. conceived this project. P.A.‐P., I.P.‐Z., and L.H. conducted LT‐STM/STS and NC‐AFM measurements. J.B. provided resources and supervision. P.A.‐P. and I.P.‐Z. performed the LT‐STM/STS and NC‐AFM data analysis. P.A.‐P., I.P.‐Z., M.C., and F.S. performed RT‐STM and ARPES measurements. P.A.‐P. and I.P.‐Z. performed the data analysis. A.E., F.S., M.C., and I.P‐Z. performed XPS measurements and data analysis. M.I., F.G., and A.G.‐L. performed the DFT calculations. M.A. and Z.A. performed EPWE simulations. M.S. and A.S. synthesized and purified the precursor molecules. P.A.‐P., M.I., D.G.O., A.G.‐L., M.C., and I.P.‐Z. contributed to writing the manuscript. All authors contributed to the revision and final discussion of the manuscript.

## Supporting information



Supporting Information

## Data Availability

The data that supports the findings of this paper are available from the corresponding authors upon request.
