# Supplementary material for: A Functional 2D Carbon Allotrope Combining Nanoporous Graphene and Biphenylene Segments
Source: Adv Mater. 2025 Dec 2;38(51):e11706. doi: 10.1002/adma.202511706 (PMC13568906; doi:10.1002/adma.202511706)
Supplement: Supplementary file 1 [file ADMA-38-e11706-s001.docx]

Supplementary Information for: A functional 2D carbon allotrope combining nanoporous graphene and biphenylene segments

Paula Angulo-Portugal^1,2†^, Martin Irizar^2,3†^, Longfeng Huang^4^, Mamun Sarker^5^, Mustafa A. Ashoush^6^, Zakaria M. Abd El-Fattah^6,7^, Johannes Barth^4^, Frederik Schiller^1^, Afaf El-Sayed^1,8^, Fei Gao^3^, Dimas G. de Oteyza^3,9^, Alexander Sinitskii^5^, Aran Garcia-Lekue^3,10*^, Martina Corso^1*^, Ignacio Piquero-Zulaica^1,10*^

1 Centro de Física de Materiales (CFM-MPC), CSIC-UPV/EHU, Manuel Lardizabal 5, 20018 San Sebastian, Spain

2 Department of Polymers and Advanced Materials: Physics, Chemistry and Technology, Faculty of Chemistry, University of the Basque Country UPV/EHU, 20018 San Sebastian, Spain

3 Donostia International Physics Center (DIPC), Manuel de Lardizabal 4, E-20018 San Sebastian, Spain

4 Physics Department E20, TUM School of Natural Sciences, Technical University of Munich, James-Franck-Straße 1, D-85748 Garching, Germany

5 Department of Chemistry and Nebraska Center for Materials and Nanoscience, University of Nebraska − Lincoln, Lincoln, Nebraska 68588, United States

6 Physics Department, Faculty of Science, Al-Azhar University, Nasr City, E-11884, Cairo, Egypt

7 Physics Department, Faculty of Science, Galala University, New Galala City, Suez, 43511, Egypt

8 Department of Electrical and Computer Engineering, University of California Los Angeles, Los Angeles, CA 90095, USA

9 Nanomaterials and Nanotechnology Research Center (CINN), CSIC-UNIOVI-PA, El Entrego, Spain

10 IKERBASQUE, Basque Foundation for Science, Plaza Euskadi 5, 48009 Bilbao, Spain

^†^ These authors contributed equally to this work

**1. On-surface synthesis of 12-pGNRs**

**Figure S1**: On-surface synthesis diagram of 12-pGNRs on Au(111). (a) On-surface synthesis steps from DBDT precursor (**1**), to DBDT-polymers (**2**) and the final 12-pGNR (**3**). The STM image and superimposed chemical model highlight the formation of DBDT-polymers (**2**) after the Ullmann-like coupling reaction at 200 °C and the tilted configuration of some phenyl rings at the edges. The combined STM and nc-AFM image shows that the 12-pGNR becomes planar (**3**) upon further annealing to 405 ºC where the cyclodehydrogenation process takes place. (b) Long-range STM image of a sub-monolayer coverage of 12-pGNRs on Au(111). All STM image parameters: I = 10 pA; V = 100 mV.

**2. Electronic property characterization of 12-pGNRs**

The molecular orbitals of 12-pGNRs are spatially inhomogeneous (Figure 2e) both in the plane of the ribbon (Figs. S2, S3) and in the vertical direction (Fig. S4), therefore the proper electronic characterization is achieved by performing point d*I*/d*V* spectroscopy (STS) at different positions of the 12-pGNR (Figure S2a). Since the 12-pGNR is grown on Au(111), the pristine Au(111) spectrum is also shown in black (Figure S2b). This spectrum shows the characteristic step at -480 mV corresponding to the Au surface state^1^. This surface state feature can still be discerned when STS is performed at the 12-pGNR backbone (green spectrum). Note that this feature appears inside the bandgap of the 12-pGNR but does not belong to the GNR (see Fig. S2c). In addition, the onset of the VB is best observed for STS taken at the nanoribbon edges. The two spectra taken at the edges (red and brown spectra) show a similar intensity for the onset of the VBs (in agreement with the -1.2 V d*I*/d*V* map of Fig. 2c) and become different for the VB-2, in agreement with the discontinuous edge conductance observed in the -1.5 V d*I*/d*V* map of Figure 2c. In addition, the onset of the CBs is well determined with STS taken at the edges (red and brown spectra) and nanopore (blue spectrum) of the 12-pGNR. These spectra are in good agreement with other STS spectra taken in a different microscope for the very same 12-pGNRs^2^.

**Figure S2**: STS measurements of 12-pGNRs on Au(111). (a) Constant-height bond-resolved (BR)-STM image acquired with a CO-functionalized tip. (b) STS spectra acquired at the positions indicated with colors in (a). Set-point parameters: I_set_ = 150 pA; V = 2 V; lock-in: V_mod_ = 30 mV; f = 403 Hz. (c) DFT calculated band structure of 12-pGNRs along the ΓX direction (i.e., along the armchair direction of the 12-pGNR).

**Figure S3**: Experimental dI/dV map comparison with DFT LDOS maps and wave function simulations. (a) cc-dI/dV maps acquired at 2 V, 1.7 V, -1.2 V and -1.5 V. (b) LDOS map simulations for the main frontier bands obtained at z = 6 Å away from the 12-pGNR. The 250 meV integration windows are indicated in (d). A reasonable agreement between experiment (a) and theory (b) is achieved. (c) Wave functions at the Γ point corresponding to the main frontier bands (i.e., from VB-2 to CB+3). Some of the wave functions are degenerate in energy. (d) DFT band structure of 12-pGNR along the ΓX direction (i.e., along the armchair direction of 12-pGNRs). dI/dV map parameters: I_set_ = 130 pA; V_mod_ = 30 mV; f = 403 Hz.

**Figure S4**: Height-dependent DFT LDOS map simulations for the main frontier bands of 12-pGNRs. (a) DFT LDOS map simulations (obtained at *z* = 4, 5 and 6 Å away from the 12-pGNR plane) for the different frontier bands. (b) 12-pGNR band structure. The 250 meV integration regions used to calculate the LDOS maps are indicated with green, yellow, purple and grey rectangles.

**Figure S5**: Series of cc dI/dV maps of 12-pGNRs acquired for the occupied region (sample bias from -2 V to -1.1 V). dI/dV map parameters: I_set_ = 130 pA; V_mod_ = 30 mV; f = 403 Hz.

**Figure S6**: Series of cc dI/dV maps of 12-pGNRs acquired for the unoccupied region (sample bias from 1.5 V to 2.2 V). dI/dV map parameters: I_set_ = 130 pA; V_mod_ = 30 mV; f = 403 Hz.

**3. Growth of 12-pGNRs and NPGs on Au surfaces, defects and their impact on the electronic structure**

Figure S7 summarizes the different substrates that we have used to grow 12-pGNRs. The Au(13 11 12) surface corresponds to a selected region of our curved Au(645) crystal with kinked steps. In particular, the Au(13 11 12) is close to the Au(111) and showed a reasonable alignment of the 12-pGNRs along the kinked steps (Figure S7b). However, on Au(788) the 12-pGNR alignment was even better, and this is where the ARPES measurements were performed (Figure 3). Note that, surprisingly, on Au(788) we encountered different manners of 12-pGNR alignment on several preparations. When 12-pGNRs aligned parallel to the steps (as in Figure S7d), some poly-*para*-phenylene (PPP) chains appeared sandwiched in between 12-pGNRs and presumably improved the alignment of the 12-pGNRs along the steps^3^. On the other hand, when no PPP chains appeared on the surface, 12-pGNRs did not align along the [1,-1,0] step direction but rather followed other crystallographic directions (as in Figure S7c). The presence of PPP chains might originate from detached phenyl rings from DBDT molecules during the on-surface synthesis process on the stepped Au(788). Curiously, we never observed any PPP chain formation on flat Au(111) (Figure S7a). A more detailed discussion of this effect is beyond the scope of this work and will be addressed in future studies.

**Figure S7**: RT-STM images for the 12-pGNRs fabricated on (a) Au(111), (b) Au(13 11 12) and (c, d) Au(788). STM parameters: (a) I = 70 pA; V = 1 V, (b) I = 102 pA; V = 1 V, (c) I = 970 pA; V = -1.2 V, (d) I = 130 pA; V = -1.2 V.

**Figure S8**: ARPES and EPWE photoemission intensity comparison for 12-pGNR bands. (a) ARPES photoemission intensity map (in second derivative) taken from Figure 3c. (b) EPWE photoemission intensity simulation (in second derivative) with the EPWE calculated band structure superimposed on top. A reasonable agreement between experiment and theory is achieved.

**Figure S9**: RT-STM, LT-STM and nc-AFM images highlighting the formation of extended NPG structures. (a, b) Long-range RT-STM images showing the successful formation of long 12-pGNRs and extended NPG structures upon lateral fusion. (c) LT-STM image of an NPG structure with 6 fused 12-pGNRs. (d) LT-STM (top) and nc-AFM (bottom) of an NPG featuring one graphene type and two biphenylene type fusions. STM parameters: (a) I = 100 pA; V = 1 V, (b) I = 0.9 nA; V = 50 mV, (c) I = 100 pA; V = 100 mV, (d) I = 100 pA; V = 50 mV.

The rearrangement or migration of the edge phenyl rings of the DBDT precursor to the neighboring carbon atoms gives rise to the most common defects observed in 12-pGNRs and NPGs. As it can be observed in the STM image and proposed chemical structure of Fig. S10a, a phenyl ring has migrated to the nearest neighboring carbon atom and protrudes to the left of the 12-pGNR. We can confirm that this phenyl ring migration is quite general for several precursors that generate chevron-type GNRs as we have already observed in our previous works^4^.

For the NPG case, the main defects encountered are summarized in Fig. S10b and c. In panel b, a phenyl ring has migrated to the nearest neighboring carbon atom (indicated by the white dashed phenyl ring) and has also fused to the right NPG, forming new C–C bonds (highlighted in red). We suggest that this scenario precludes the proper fusion of both edges together into a wider single NPG. On the other hand, in panel c, a phenyl ring is missing (outlined with white dashed lines), inducing a distortion of the [18]annulene nanopore.

This phenyl ring migration or absence can induce the irregular appearance of some edges or nanopores. However, considering that the electronic properties of these NPGs are governed by the chevron nanoribbon segments created in between the nanopores and these chevron nanoribbons are weakly coupled, such local distortions at the edges or nanopores should have minimal impact on the global electronic properties of the extended NPG structures. Such phenyl migration was also observed and used to synthesize [18]annulene pores at the edges of 7-13-aGNRs and NPGs. This edge transformation did not affect the frontier bands as observed with LT-STS and DFT simulations^5^.

**Figure S10**: Summary of the most common defects encountered in 12-pGNRs and NPG structures. On the top, STM images and on the bottom, corresponding superimposed chemical structures. (a) A phenyl ring from the edge of the DBDT precursor has migrated to the neighboring carbon atom. As a result, it protrudes laterally on the 12-pGNR and the [18]annulene nanopore is not closed. (b) A phenyl ring has migrated to a neighboring carbon atom from the left NPG. In addition, this phenyl ring has fused to the right NPG. (c) A phenyl ring is missing, forming a distorted nanopore.

**Figure S11**: LT-STM and nc-AFM summary of the six fusing configurations that lead into the formation of distinct graphene type or biphenylene type NPGs on the Au(111) surface. (a) Graphene type NPGs with the three possible fusing configurations. STM parameters from left to right: (I = 100 pA; V = 50 mV), (I = 10 pA; V = 50 mV) and (I = 100 pA; V = 100 mV). (b) Biphenylene type NPGs with the three possible fusing configurations. STM parameters from left to right: (I = 10 pA; V = 50 mV), (I = 100 pA; V = 50 mV) and (I = 10 pA; V = 50 mV). Note that these six fusing configurations yield the formation of four distinct NPGs, as highlighted in Figure S12.

**Figure S12**: Schematic models of the different types of NPGs formed on Au(111). The six different fusing configurations shown in Figure S11 lead to four distinct NPG structures. (a) The aligned graphene type NPG where the nanopores are in a head-to-head configuration. (b) Two shifted graphene type NPGs are depicted. The nanopores of one 12-pGNR are shifted with respect to next fused 12-pGNR. Note that both shifted NPG-Gs are identical in terms of structural and electronic properties. (c) The aligned biphenylene type NPG-BP where the nanopores are in a head-to-head configuration. (d) Two shifted biphenylene type NPGs are depicted. Note that both shifted NPG-BPs are identical in terms of structural and electronic properties.

As it can be observed in Figure S13a, the NPG-BP VB wave function is clearly different to the 12-pGNR VB and VB-1, but resembles the VB-2 wave function of 12-pGNR instead (Figure S13b), suggesting that such biphenylenic coupling pattern upon c-BPR formation has a stronger stabilizing effect on the VB-2 than on the frontier bands.

**Figure S13**: DFT calculated wave functions at the Γ point for the (a) NPG-BP VB as compared to the (b) 12-pGNR VB, VB-1 and VB-2. The NPG-BP VB resembles the VB-2 of 12-pGNR.

**Figure S14**: DFT calculated band structure and frontier band wave functions (at the Γ point) for the four different NPGs described in Figure S12. The aligned NPG-G (a) and shifted NPG-G (b) are shown on the top panels, while the aligned NPG-BP (c) and shifted NPG-BP (d) examples are depicted on the bottom panels. While graphene-type NPGs show a direct bandgap, the biphenylene-type NPGs exhibit an indirect transition.

**4. Extended hybrid NPG-G-BP structures and electronic properties**

We have simulated the electronic structure of hybrid NPGs that emerge by alternating graphene and biphenylene fusion between 12-pGNRs in a one-to-one ratio. In particular, two possible configurations in which the pores are aligned (Figure S15a) or shifted (Figure S15c) have been studied. The DFT calculations are carried out using the same computational parameters as described in the Methods section of the main text. The corresponding band structures are shown in Figures S15b and S15d, where the blue and green color of the lines represent the contribution from the chevron biphenylene ribbon (c-BPR) and chevron graphene ribbon (c-GNR) segments, respectively. We observe that both hybrid NPG structures exhibit an indirect bandgap of ~1eV, defined by electronic bands with a dominant contribution from c-BPR (see red arrows in Figures S15b, d). On the other hand, we find that the highest occupied band at Γ is dominated by the c-GNR segment for both the aligned and shifted hybrid NPGs.

**Figure S15**: Electronic band structure of hybrid NPGs. (a) Structural model of a hybrid and aligned NPG structure. (b) Electronic band structure for the aligned NPG with projection on chevron biphenylene ribbon (c-BPR) segments in blue (left) and chevron GNR (c-GNR) segments in green (right). (c) Structural model of a hybrid and shifted NPG structure. The nanopores after the biphenylene segment are shifted or out of phase. (d) Electronic band structure for the shifted NPG with projection on c-BPR segments in blue (left) and c-GNR segments in green (right). Note that, to allow for a more direct comparison with the band structures reported in the main text, we have used an orthogonal unit cell containing four 12-pGNRs.

**5. Mechanical properties of NPG-G and NPG-BP**

Figure S16 shows the stress–strain curves for NPG-G and NPG-BP, which have been evaluated through a series of DFT calculations using the same computational parameters as described in the Methods section of the main text. The uniaxial stress-strain behavior has been examined for NPGs elongated along the growth direction of the parent 12-pGNRs (X, armchair direction) and along the perpendicular direction (Y, zigzag direction). To obtain the equivalent stress from supercell stress calculations, we have followed the methodology by F. Liu *et al.*^6^.

Our results reveal that both NPG-G and NPG-BP exhibit elastic behavior at low strain values (<2%), but the relation between strain and stress becomes nonlinear for larger strain values (see Fig. S16a, b). Moreover, we observe that, while in the non-linear regime both NPG structures exhibit distinct stress-strain curves along X and Y directions, the anisotropy is significantly quenched for NPG-BP in the linear regime. To quantify this effect, we have estimated the Young’s moduli along X and Y by linear fitting the stress-strain curves for strain levels below 2%. As summarized in the Table of Fig. S16c, NPG-BP shows a very similar Young’s moduli in both directions (~534GPa). On the other hand, NPG-G exhibits a remarkably larger Young’s modulus in the X direction (~623.3GPa) than in the Y direction (~549.8GPa), indicating that NPG-G is more resistant to elastic deformation along the direction of the parent nanoribbons. Note that the Young’s moduli reported here are in the range of previously reported values for NPG structures^7,8^, being all well below the Young’s modulus of pristine graphene^9^ (~1TPa) but superior to other 2D materials (e.g., MoS_2_^10^ and MXenes^11^). In summary, our results confirm that the nanopores reduce the stiffness as compared to graphene and demonstrate that NPG-G exhibits strongly anisotropic mechanical properties for all strain levels. More interestingly, they also reveal that including biphenylene groups in the carbon network drastically reduces the anisotropy in the linear regime.

**Figure S16**: Mechanical properties of NPG-G and NPG-BP. (a) Structural model (top) and stress-strain curve (bottom) of NPG-G. (b) Structural model (top) and stress-strain curve (bottom) of NPG-BP. (c) Table summarizing the obtained Young’s moduli for NPG-G and NPG-BP along the X and Y directions.

**6. Stability of 12-pGNRs under O_2_ exposure**

**Figure S17**: LT-STM, nc-AFM and cc-dI/dV maps for a 12-pGNR after its exposure to 450 L of O_2_ in UHV (sample kept at RT). (a, b) STM image of a 12-pGNR and its corresponding nc-AFM image. STM parameters: I = 10 pA; V = 50 mV. (c, d) cc-dI/dV maps acquired at -1.5 V and 1.9 V respectively. The structural and electronic features remain the same as the unexposed 12-pGNRs (see Figures S5 and S6). dI/dV parameters: I_set_ =130 pA; V_mod_ = 30 mV; f = 403 Hz.

**7. Air stability of 12-pGNRs and NPGs**

In order to test the stability of 12-pGNRs and NPGs in air, we prepared one sample that combines both materials as shown in Figure S18. We have then characterized each step of air exposure and the recovery process (in UHV via thermal annealing steps at 400 °C and 510 °C) with lab-based X-ray photoelectron spectroscopy (XPS, with Al K_α_ line at 1486.6 eV) and RT-STM. First, we characterized the as-prepared sample (blue spectra in XPS and STM image) and observed the C 1s core level peak featuring the expected C-H and C-C component ratios (Fig. S18a, left). This is consistent with the mixed 12-pGNR and NPG structures shown in the blue STM image (Fig. S18b, bottom). The coverage was estimated to be ≈0.75 monolayers (MLs). No oxygen peak was detected (panel a, right). Then, we have exposed our sample to air for 1 hour, a duration substantially longer than in previous air exposure experiments performed for similar molecular nanostructures on Au(111)^12^. After air exposure, the sample was again inserted in the UHV system. Under these conditions STM cannot be easily performed but XPS shows an increase of the C 1s peak (≈1.40 ML) and the emergence of the O 1s peak (black spectra in panel a). This peak likely originates from the adsorption of water, CO_2_, fatty acids and volatile organic compounds on the sample, and amounts to ≈0.15 ML. After annealing the sample at 400 ºC in UHV for 10 min, the STM image (in red) shows that the NPGs and 12-pGNRs are mostly intact and appear surrounded by some additional residual contamination. XPS indicates that the C 1s peak (red spectrum) is slightly larger than the initial case (≈0.88 ML) and the O 1s peak is drastically reduced (≈0.05 ML) with respect to the analysis after air exposure and shifts to lower binding energies, implying still the chemical bonding of spurious amount of oxygen to the material or other remaining organic compounds. Further annealing to 510 ºC in UHV for 2 min does not alter the C 1s peak (≈0.88 ML) (green spectrum, panel a), confirming that the 12-pGNRs and NPGs remain robust (as seen in the green STM image of panel b, top). The O 1s peak cannot be completely suppressed, but its intensity remains very low (≈0.04 ML) (considering that at this X-ray photon energy, the photoionization scattering cross section of O 1s is almost three times larger than the one of C 1s). In summary, even though some small oxygen doping cannot be fully discarded, this experiment demonstrates that both 12-pGNRs and NPGs are mostly stable upon air exposure and should thus be transferable into devices following previously reported transfer protocols from Au films to oxides^13–16^.

**Figure S18**: Air exposure (for 1h) of 12-pGNRs and NPGs. (a) XPS spectra for the C 1s (left) and O 1s (right) components for the different stages of the air exposure experiment: as prepared (blue), after air exposure (black), after annealing to 400 °C in UHV (red) and after annealing to 510 °C in UHV (green). The estimated C and O coverages are indicated in MLs. (b) RT-STM images obtained for the as prepared sample (blue), after annealing to 400 °C in UHV (red) and after annealing to 510 °C in UHV (green). STM parameters from top to bottom: *V* = 1 V, *I* = 150 pA (top), *V* = 1 V, *I* = 150 pA (middle) and *V* = 500 mV, *I* = 100 pA (bottom).

**References**:

1.	Li, J. *et al.* An organic array of quantum corrals modulated by the gold herringbone electronic superlattice. *Nanoscale* **17**, 10314–10323 (2025).

2.	Fan, Q. *et al.* Bottom-up Synthesis and Characterization of Porous 12-Atom-Wide Armchair Graphene Nanoribbons. *Nano Lett.* **24**, 10718–10723 (2024).

3.	Liu, M. *et al.* Graphene-like nanoribbons periodically embedded with four- and eight-membered rings. *Nat Commun* **8**, 14924 (2017).

4.	Costa, P. S., Teeter, J. D., Enders, A. & Sinitskii, A. Chevron-based graphene nanoribbon heterojunctions: Localized effects of lateral extension and structural defects on electronic properties. *Carbon* **134**, 310–315 (2018).

5.	Moreno, C. *et al.* On-surface synthesis of porous graphene nanoribbons mediated by phenyl migration. *Commun Chem* **7**, 219 (2024).

6.	Liu, F., Ming, P. & Li, J. Ab initio calculation of ideal strength and phonon instability of graphene under tension. *Phys. Rev. B* **76**, 064120 (2007).

7.	Mortazavi, B. *et al.* Nanoporous graphene: A 2D semiconductor with anisotropic mechanical, optical and thermal conduction properties. *Carbon* **147**, 377–384 (2019).

8.	Han, T. *et al.* Tuning the mechanical properties of nanoporous graphene: a molecular dynamics study. *Mater. Res. Express* **6**, 095619 (2019).

9.	Lee, C., Wei, X., Kysar, J. W. & Hone, J. Measurement of the Elastic Properties and Intrinsic Strength of Monolayer Graphene. *Science* **321**, 385–388 (2008).

10.	Bertolazzi, S., Brivio, J. & Kis, A. Stretching and Breaking of Ultrathin MoS2. *ACS Nano* **5**, 9703–9709 (2011).

11.	Lipatov, A. *et al.* Elastic properties of 2D Ti3C2Tx MXene monolayers and bilayers. *Science Advances* **4**, eaat0491 (2018).

12.	Gómez-Fernández, J. M. *et al.* Influence of the substrate in the on-surface synthesis and air stability of 1D metal-organic oligomers. *Applied Surface Science* **693**, 162804 (2025).

13.	Llinas, J. P. *et al.* Short-channel field-effect transistors with 9-atom and 13-atom wide graphene nanoribbons. *Nat Commun* **8**, 633 (2017).

14.	Mutlu, Z. *et al.* Bottom-Up Synthesized Nanoporous Graphene Transistors. *Advanced Functional Materials* **31**, 2103798 (2021).

15.	Borin Barin, G. *et al.* Surface-Synthesized Graphene Nanoribbons for Room Temperature Switching Devices: Substrate Transfer and ex Situ Characterization. *ACS Appl. Nano Mater.* **2**, 2184–2192 (2019).

16.	Moreno, C. *et al.* Bottom-up synthesis of multifunctional nanoporous graphene. *Science* **360**, 199–203 (2018).
